# Concurrent-Mode CMOS Detector IC for Sub-Terahertz Imaging System

**DOI:** 10.3390/s22051753

**Published:** 2022-02-23

**Authors:** Moon-Jeong Lee, Ha-Neul Lee, Ga-Eun Lee, Seong-Tae Han, Jong-Ryul Yang

**Affiliations:** 1 Department of Electronic Engineering, Yeungnam University, Gyeongsan 38541, Korea; hsg03170@yu.ac.kr (M.-J.L.); gksmf4461@yu.ac.kr (H.-N.L.); gaeun@ynu.ac.kr (G.-E.L.); 2 Electrophysics Research Center, Korea Electrotechnology Research Institute, Changwon 51543, Korea; saiph@keri.re.kr

**Keywords:** CMOS detector, concurrent-mode, differential detector IC, imaging SNR, integrated folded-dipole antenna, sub-terahertz imaging, voltage responsivity

## Abstract

A CMOS detector with a concurrent mode for high-quality images in the sub-terahertz region has been proposed. The detector improves output-signal coupling characteristics at the output node. A cross-coupling capacitor is added to isolate the DC bias between the drain and gate. The detector is designed to combine a 180° phase shift based on common source operation and an in-phase output signal based on the drain input. The circuit layout and phase shift occurring in the cross-coupled capacitor during phase coupling are verified using an EM simulation. The detector is fabricated using the TSMC 0.25-μm mixed-signal 1-poly 5-metal layer CMOS process, where the size, including the pad, is 1.13 mm × 0.74 mm. The detector IC comprises a folded dipole antenna, the proposed detector, a preamplifier, and a voltage buffer. Measurement results using a 200-GHz gyrotron source demonstrate that the proposed detector voltage responsivity is 14.13 MV/W with a noise-equivalent power of 34.42 pW/√Hz. The high detection performance helps resolve the 2-mm line width. The proposed detector exhibits a signal-to-noise ratio of 49 dB with regard to the THz imaging performance, which is 9 dB higher than that of the previous CMOS detector core circuits with gate-drain capacitors.

## 1. Introduction

Terahertz (THz) waves represent frequencies of the 0.1–10 THz bands of the frequency spectrum. Located between radio and light waves, THz waves with high transmittance and directivity are expected to be widely used in the fields of security imaging, radio astronomy, medical imaging, and art [1,2,3,4,5,6,7]. A THz imaging system is capable of nondestructive inspection owing to the low ionization energy with regard to frequency. In recent times, the THz wave has been of interest as a frequency resource to replace the millimeter wave, as it can be used in communication beyond the limit of short-wavelength waves [8].

Unlike millimeter wave infrastructure, an active full-wave structure is required to develop a safe and high-resolution THz imaging system. The performance of an active system can be expressed by the signal-to-noise ratio (SNR), in which a measurement target is independently placed between a transmitter and a receiver, and the receiver detects and outputs the signal reflected or transmitted from the target. The SNR of the image quality factor can be calculated as the ratio between the output signals when the transmitted signal is reflected away by a metal target and when the transmitted signal passes through a nonreflective target; the reflected transmission output measures the noise of the detector. The image quality of the output can be measured using high-power sources; however, the implementation of the high-powered transmitter at the THz frequency band is difficult and expensive to fabricate. A receiver with high sensitivity characteristics improves the performance of an imaging system. The sensitivity of the receiver is measured based on voltage responsivity (*R_V_*) and noise-equivalent power (*NEP*). *R_V_* represents the output voltage magnitude as a function of the input signal power, and *NEP* demonstrates the noise characteristics of the detector. A receiver with low *NEP* exhibits a smaller power threshold to distinguish between signal and noise, allowing for more sensitive operation. High *R_V_* detectors can minimize *NEP*, resulting in high performance [9].

The surface plasma phenomenon of field-effect transistors, which was first introduced by M. Dyakonov and M. Shur, exhibited potential for sensing in the THz band using a standard complementary metal-oxide-semiconductor (CMOS) process [10,11]. A CMOS detector capable of detecting a signal above the operating frequency is known as a self-mixing detector or a square row detector, and outputs a direct current (DC) voltage [12]. Body bias control can modify the electrical characteristics of the detector core to improve the performance by changing the subthreshold slope [13]. In addition, a CMOS detector with body bias control has demonstrated a wide dynamic range with strong voltage responsivity [13]. Detector bias changes the electric field effect of the detector, which requires various operating parameters. Cascode topology allows a detector to operate similar to a general amplifier, where the source, gate, and drain interfaces are simplified, thereby significantly improving the detector performance [14,15,16,17,18]. CMOS detector topologies have been proposed with commonly used gate and drain input structures, where the phase is determined by adding transconductance terms using Taylor expansion [19]. The analysis confirmed that detection performance can be improved based on the differential phase input with drain bias. A detector circuit with weak inversion mode operation characteristics can help improve performance through the phase coupling of the output signal and unbiased drain.

In this study, a concurrent-mode CMOS detector integrated circuit (IC) with phase-coupled operation at the output node with input signals routing through cross-coupled capacitors is proposed. The proposed detector IC comprises a differential-folded dipole antenna, the proposed differential detector core, a pre-amplifier to convert a signal from a differential to single-ended mode, and a voltage buffer with low gain. The performance of the detector can be improved by combining the two signals of the dual detection output in one core circuit; as a result, the proposed IC functions as a high-quality THz imaging system. Section 2 describes the core configuration and operating principle of the CMOS concurrent-mode detector with phase combination. A phase difference occurs between the gate of the input node and the drain of the output node owing to the cross-coupled capacitors. The proposed detector IC was implemented using the TSMC 0.25-μm CMOS 1-poly 5-metal (1P5M) process. Section 3 presents the measurement results of the performance of the detector IC and results obtained using 200-GHz raster-scanned imaging. Section 4 concludes the study.

## 2. Proposed Detector Circuit

**Figure 1:**
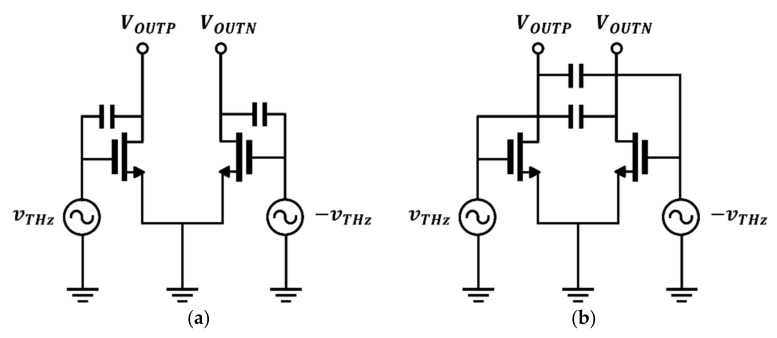
CMOS detector configuration: (**a**) Previous detector circuit with gate-drain capacitors; (**b**) proposed concurrent-mode detector.

The detector circuit with a gate-drain capacitor used to enhance the potential difference between the drain and source terminals is shown in Figure 1a. Extra gate-drain capacitors are added to a CMOS-based single gate input circuit and exhibit a high potential difference. Despite its high performance, the previous topology is sensitive to the capacitor size, making it difficult to guarantee operational safety. The limitations of the process model and layout used to optimize the capacitor size can prevent the detector from achieving optimal performance. A high-performance concurrent-mode detector circuit with safe operation via cross-coupled structure for dual operation has been proposed.

### 2.1. Proposed Detector Core Configuration

**Figure 2:**
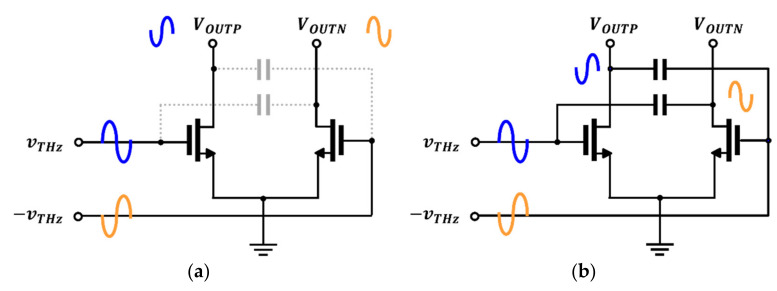
Two operations in the proposed circuit: (**a**) gate input topology; (**b**) drain input topology.

The proposed detector with a concurrent-mode operation circuit, as shown in Figure 1b, improves the quality of the THz imaging system; the circuit operates based on the square root detection of the input power and outputs a voltage signal. The proposed detector consists of the same components as the detector circuit with gate-drain capacitors, except for cross-coupled capacitors. The cross-coupled capacitors were used to transmit the THz signal to the drain of an adjacent detection transistor. Unlike the gate-drain capacitor circuit, which improves the drain-source potential difference, the capacitor blocks the gate input DC bias and only transmits the in-phase signal. The detector structure can be interpreted as a combination of the gate input and drain input circuits. The proposed detector has two operation modes. One of the operation modes of the proposed detector can be analyzed in the same method as the general common-source stage amplifier shown in Figure 2a. The output signal is inverted from the input signal, with a 180° difference.

Figure 2b shows drain input detection operation using cross capacitors connected to the drain nodes of the two transistors. The output signal of the drain input circuit exhibits the same phase as the gate input without any phase shift and is, therefore, phase-coupled to the output drain terminal. Both detector inputs include gate bias for weak inversion operation at the gate node. The operating principle of the proposed detector is defined as the concurrent-mode operation, in which an incident signal is simultaneously applied to the gate and drain of the single detector core. The concurrent-mode operating characteristics are designed using cross-coupling capacitors; a sufficient capacitance is required for high-frequency coupling. The same phase at the final output node is important for combining the two detector outputs. The proposed detector circuit was designed to incorporate the cross-coupled capacitor layout and additional core circuit phase shifts and was validated using electromagnetic simulation performed using the Keysight Advanced Design System software.

**Figure 3:**
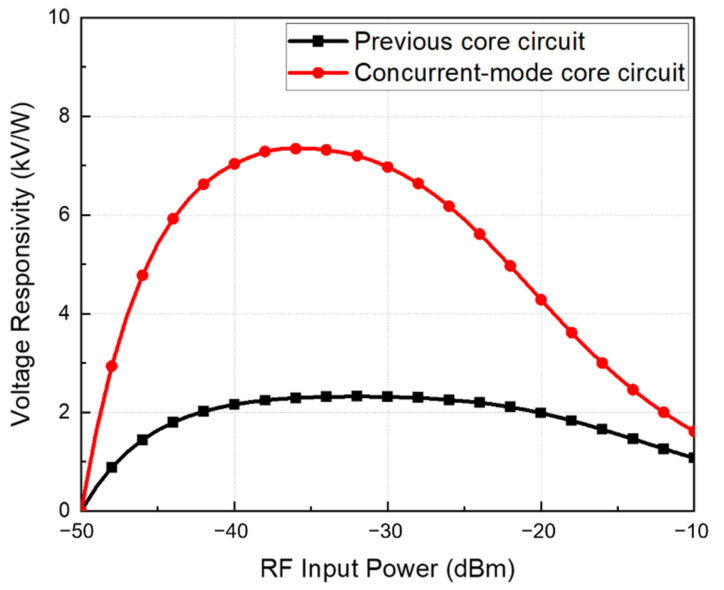
Simulated voltage responsivity of the detector core with gate-drain capacitors and the proposed concurrent-mode detector core.

The performance of the two detector cores, as shown in Figure 1, was simulated using Cadence Spectre. Although achieving the impedance-matching condition is essential for optimizing detector characteristics, two detector cores were used as transistors of the same size to compare the performance based on the detector configuration. Figure 3 shows the simulated voltage responsivities between the previous and proposed detector cores. The proposed concurrent-mode detector core exhibited a voltage responsivity of 1.5–3.3 times higher than that of the previous detector core based on the input power. The performance improvement owing to phase coupling is confirmed above the power level at which the two detection operation outputs are significant. However, beyond a certain power level, a sufficiently large power signal is incident on the gate node, and the output of the gate input circuit becomes dominant, decreasing the performance difference.

### 2.2. Folded Dipole Antenna

**Figure 4:**
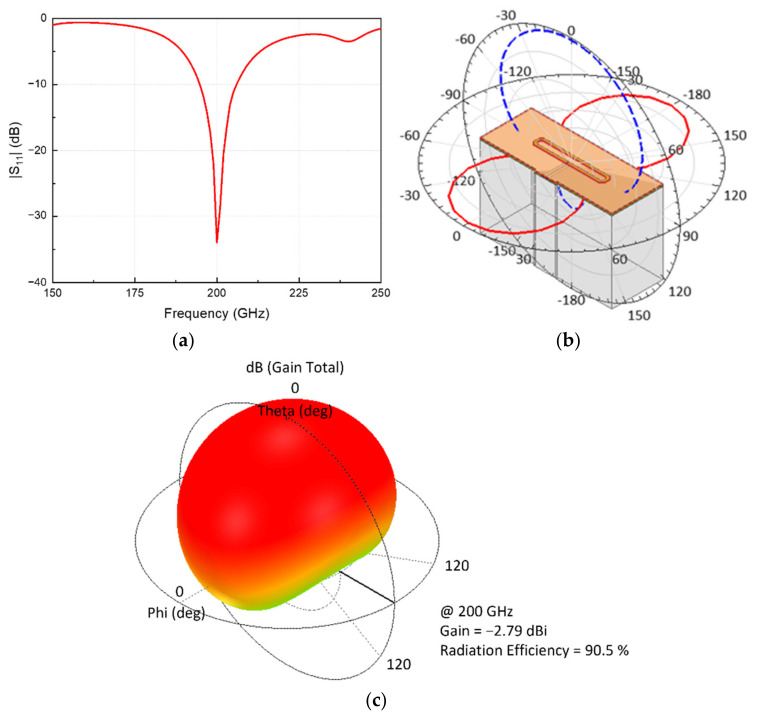
Simulation results of the integrated folded dipole antenna: (**a**) reflection coefficient |S_11_|; (**b**) far-field radiation pattern at 200 GHz; (**c**) 3D radiation pattern at 200 GHz.

A non-frequency selective detection circuit is determined for operating frequencies using an integrated antenna. A differential integrated antenna with an operating frequency of 200 GHz was designed to obtain input signals whose frequencies are higher than the device operating frequency. A grounded guard ring was placed at a sufficient distance from the antenna metal to focus on the internal electric field. The total area of the antenna, including the guard ring, is 500 μm × 200 μm. Compared with a patch antenna, the proposed antenna has a smaller area and exhibits similar performance, which is advantageous for a large-scale array. The on-chip antenna was configured as a folded dipole antenna to assume the operational mode of the detector circuit by applying a gate bias through a virtual ground. The radiating metal was folded at 45° to balance the paths of the inner and outer lengths. Chamfered radiating metal edges can prevent distortion from the antenna owing to processing changes. The simulation results using the 3-D EM simulation tool, ANSYS Electronics, demonstrate 11 GHz of −10 dB bandwidth corresponding to 195–206 GHz, as depicted in Figure 4a. The simulation data in Figure 4b represent the E-field and H-field characteristics in the far field. As shown in Figure 4c, the antenna radiation gain is simulated at −2.79 dBi at 200 GHz, exhibiting a peak radiation efficiency of 90.5%. In the far field, the simulated data confirm that the antenna performance is omnidirectional and suitable for image measurement.

### 2.3. CMOS Detector IC Implementation

**Figure 5:**
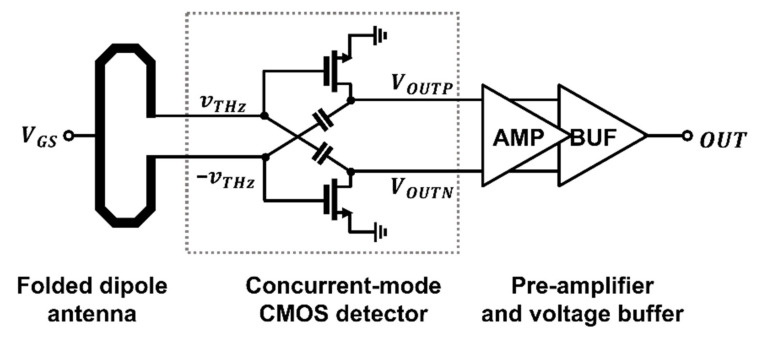
CMOS detector IC comprising a 200-GHz folded dipole antenna, the proposed detector, a differential-to-single-ended preamplifier, and a voltage buffer.

The CMOS detector IC, including the in-phase coupled detector, is illustrated in Figure 5. The final output signal was generated using a differential-to-single-ended preamplifier and monitored using an impedance-converting voltage buffer. A validated preamplifier and voltage buffer used the same circuit to compare the inherent detector core circuits [19].

**Figure 6:**
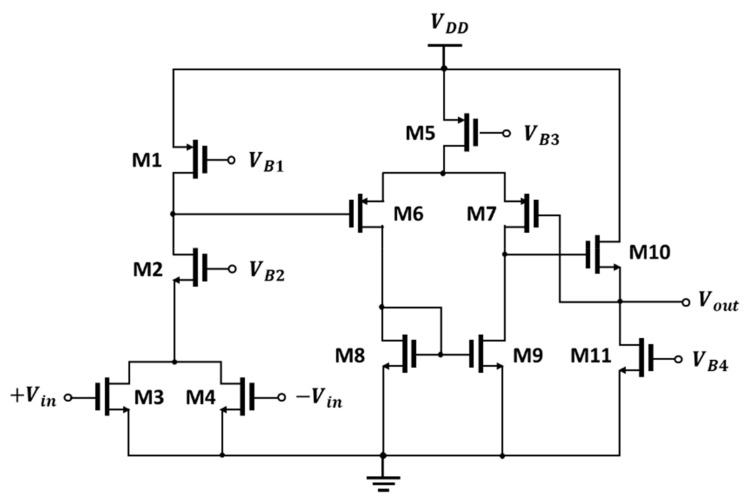
Schematic of the preamplifier with a voltage buffer.

Figure 6 shows the circuit used to monitor the output. Voltages *V_B_*_1_, *V_B_*_2_, *V_B_*_3_, and *V_B_*_4_ are biased in the circuit based on a current reference circuit. A common gate transistor, M2, acting as a level shifter and isolator, was used to change the output voltage of the transconductance stage at the load. The output signals of the detector core were transferred to transistors M3 and M4, which operated in the subthreshold region by self-biasing to the DC output voltage of the core. The converted DC and coupled sub-terahertz signals at terminals *V_OUTP_* and *V_OUTN_* are combined as currents at the drain node of M3 and M4. In the preamplifier, the in-phase DC signals are summed and the out-of-phase sub-terahertz signals are canceled [2]. In this operation, the differential input is converted into a single-ended output at the detector IC. The output of the preamplifier with unity gain is connected to M7 of the source follower via negative feedback for operational stability to output the final signal.

**Figure 7:**
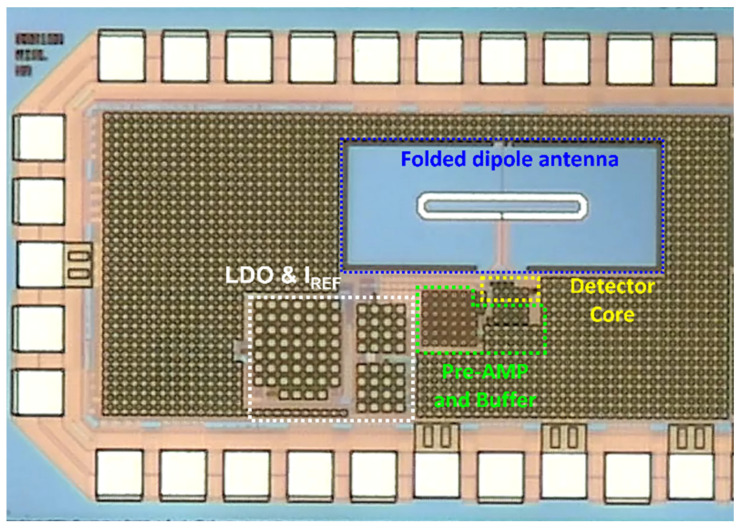
Die photograph of the proposed detector IC implemented using the TSMC 0.25-μm mixed-signal CMOS process comprising a 200-GHz folded dipole antenna, the proposed detector core, a pre-amplifier, and a voltage buffer.

The proposed detector IC is implemented using the TSMC 0.25-μm mixed-signal process, as shown in Figure 7. The gate bias of the detector core is applied to the alternating current (AC) ground node of the folded dipole antenna using an external instrument. The additional isolation was provided by the integrated resistance of 60 kΩ between the node and an I/O pad. Bias voltages except the gate voltage were provided by integrated low-dropout (LDO) regulator and current reference (I_REF_) circuits to ensure the operational safety of the proposed detector. The size of the fabricated chip including the pad and proposed detector circuit is 1.13 mm × 0.74 mm. The difference between the two differential output DC voltages and bias voltage offset each other, and signal leakage into the output signal is attenuated by a radio-frequency (RF) choke at the input transistors of the preamplifier; consequently, the differential input is converted to a single-ended output. The detected signal is delivered to the final output pad using a voltage buffer with a gain of −1.5 dB, which determines the detector performance based on impedance conversion.

## 3. Measurement Results and Discussions

### 3.1. Measurement Setup

**Figure 8:**
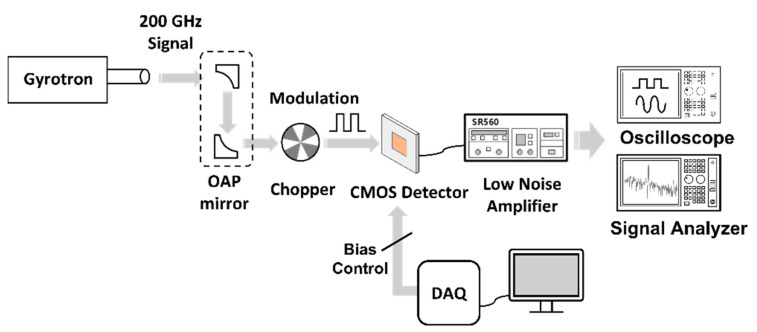
Measurement setup to analyze the performance of the proposed detector IC. An oscilloscope to measure *R_V_* and a signal analyzer to obtain noise-equivalent power have been used.

The setup used to measure the performance of the proposed detector is illustrated in Figure 8. The 200-GHz signal generated by the gyrotron source demonstrates Gaussian beam characteristics, whose focal plane is aligned by off-axis parabolic (OAP) mirrors [20] with a focal length of 15.24 cm. A physical chopper located in the focal plane reduces the flicker noise by transmitting the detected output DC voltage along with an AC signal. The measurements were conducted with a modulation frequency of 200 Hz for avoiding the effect of switching noise from the power supply. To monitor the constant voltage output of the LDO regulator, digital 4-bit control signals were applied using the National Instruments (NI) data acquisition board (DAQ). Before reaching the monitoring equipment, the detector IC output was amplified with a gain of 5 through a bandpass filter of 100–300 Hz using an SR560 low-noise voltage amplifier manufactured by Stanford Research Systems. *R_V_* was measured using an oscilloscope, and *NEP* was measured using the Keysight N9010B signal analyzer. The unamplified detector performance was measured by dividing the measurement with the amplifier gain.

**Figure 9:**
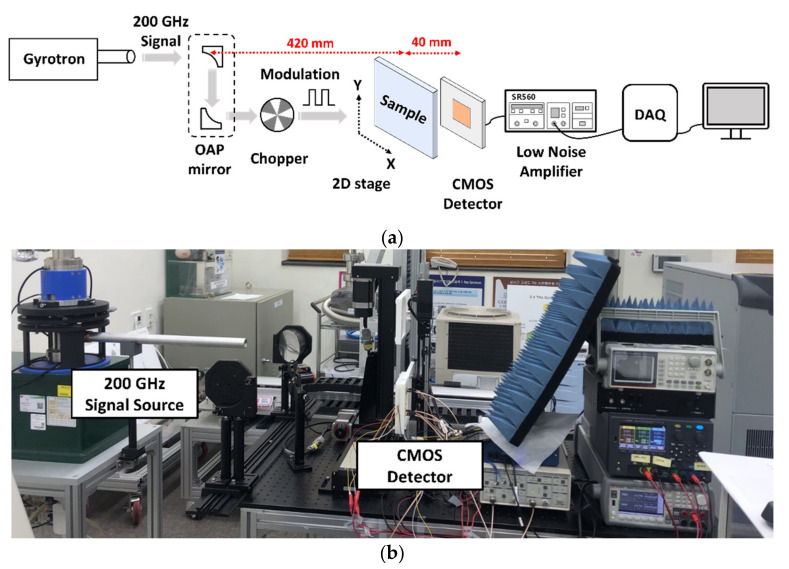
Measurement setup: (**a**) block diagram for THz imaging using raster scanning; (**b**) experimental setup.

In the THz imaging system test, as shown in Figure 9a, the distance between the mirror and the sample was 420 mm, and that between the sample and the proposed CMOS detector IC was 40 mm; the sample was placed on the XY stage. During image acquisition, the DAQ generated a digital 4-bit control code and analyzed the measurement data. The output signals were acquired using the NI DAQ hardware and NI LabVIEW software tools. The sample was moved at intervals of 1 mm in the measurement environment, as shown in Figure 9b, and the final output image was obtained using 2-D raster scanning.

### 3.2. Proposed Detector IC Performance

The performance of sub-THz CMOS detectors is determined by *R_V_* and *NEP*. *R_V_* is defined as the change in the output voltage based on the presence or absence of the incident signal with the specific power applied to the detector; it is calculated as:

(1) $$R_{V}=\frac{V_{OUT}-V_{DCOFF}}{P_{IN}}=\frac{V_{OUT}-V_{DCOFF}}{P_{D}\cdot A_{EFF}}\;\;\left[V/W\right],$$

where *V_OUT_* is the output voltage when the input power *P_IN_* is applied to the detector, *V_DCOFF_* is the output voltage without the incident signal, *P_D_* is the power density incident to the detector IC, and *A_EFF_* is the effective antenna area, which includes the integrated antenna and wavelength characteristics [9,13]. *NEP* is defined as the input power level that becomes equal to the noise generated from the detector itself; it is expressed using *R_V_* as:

(2) $$NEP=\frac{\sqrt{N_{V}}}{R_{V}}\left[W/\sqrt{Hz}\right],$$

where *N_V_* denotes the noise spectral density [9].

**Figure 10:**
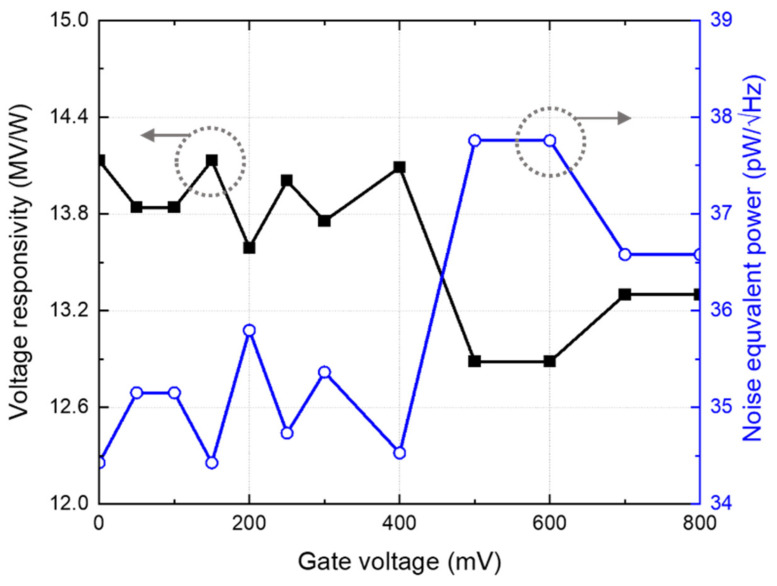
Measurement results of the voltage responsivity and the noise-equivalent power using the proposed CMOS detector IC at 200 GHz.

When using a receiver antenna to transmit a THz signal to a detector, it is vital to determine the characteristics of the detector antenna and consider the input power equation of the detector to accurately analyze and measure the detector performance. The unit power density of the gyrotron measured at the detector position is 0.5 W/m^2^, and the effective area considering the receiving antenna gain at 200 GHz is 9.62 × 10^−8^ m^2^ [13]. The input power was calculated based on the measured performance, considering the difference in antenna gain according to the radiation area and similar antenna simulation values [21]. Figure 10 shows the measured *R_V_* and *NEP* values of the proposed CMOS detector with different gate bias voltages. The results exhibit high *R_V_* and low noise when the gate bias is lower than the threshold voltage. *R_V_* and *NEP* were 14.13 MV/W and 34.42 pW/√Hz, respectively, under the gate bias condition of 150 mV. Table 1 lists the performance comparison of the proposed CMOS detector with previously developed detector core configurations. The proposed CMOS detector exhibits higher *R_V_* compared with the other detectors. As a result of comparing detectors with the same minimum gate length, the proposed detector showed the lowest *NEP* and highest *R_V_*. In previous studies, while calculating the effective area of an antenna, the difference in the radiation area between the antenna simulations and measurements was not considered. The performance of the proposed detector was calculated using the simulation data of the integrated antenna, which includes the ground guard ring in the simulation model for providing the same radiation area as the fabricated IC.

### 3.3. Images Obtained Using the Proposed Detector IC

**Figure 11:**
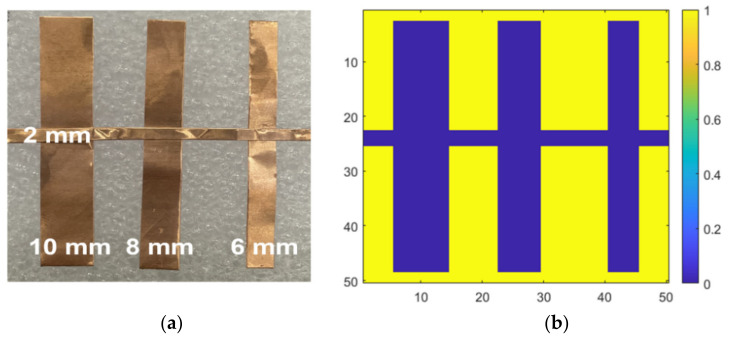
Sample target for sub-THz imaging: (**a**) photograph of a sample with different copper widths; (**b**) digitalized image of the sample to image correlation.

**Figure 12:**
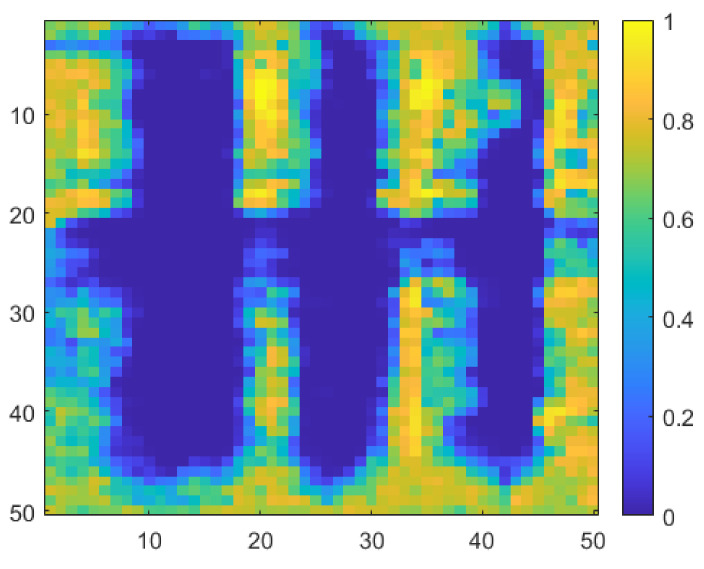
Measurement image using the proposed CMOS detector IC.

Copper foil tapes of different thicknesses were placed on the Styrofoam substrate to measure the resolution of the proposed detector, as shown in Figure 11a. The sample size was 50 mm × 50 mm. Considering that the wavelength of 200 GHz in the air is 1.5 mm, the sample was manufactured considering a thickness of ≥2 mm. The real sample was digitized, as shown in Figure 11b, for a digital area comparison using MATLAB. The sub-THz imaging at 200 GHz yielded results that are 63.6% identical to those of the digitalized sample. As shown in Figure 12, imaging results obtained using the proposed detector demonstrated that a 2-mm thick conductive target could be distinguished from the background. The measurement image is more distributed than the physical sample, as the passing waves are dispersed over the distance of 40 mm between the detector and sample. Considering the difficulty in identifying the wavelength width of the metal using a CMOS detector owing to the distance between the sample and detector, the measurement result exhibits a high-resolution image. All the images were compared by normalizing them to either maximum or minimum ratios.

**Figure 13:**
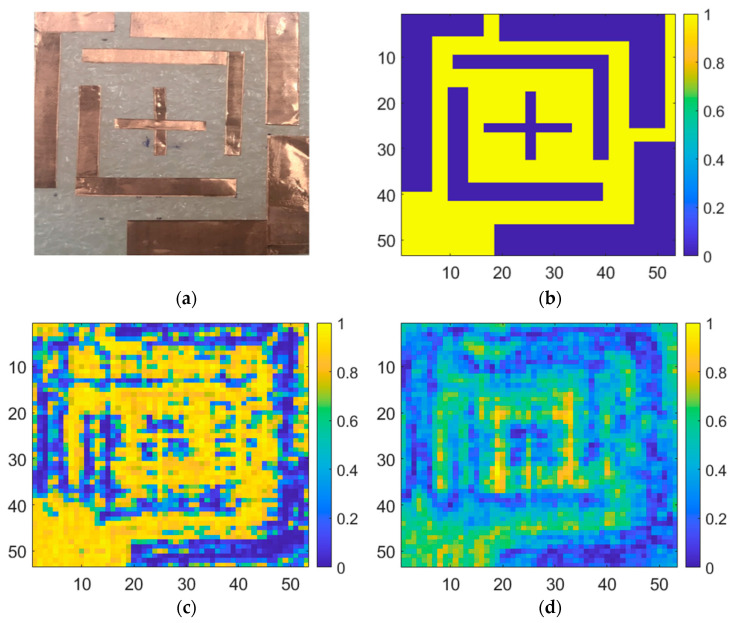
Sub-THz imaging in the previous study [23]. (**a**) Photograph of a real sample; (**b**) digitized image; (**c**) using a common-source detector circuit with the standard capacitors in the process design kit; (**d**) using a common-source detector circuit with the customized capacitors.

**Figure 14:**
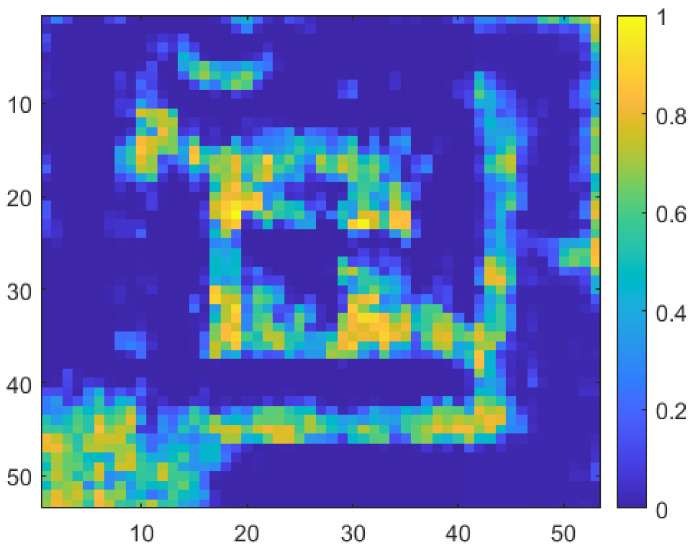
Imaging measurement using the proposed concurrent-mode detector at 200 GHz.

As illustrated in Figure 13a, the sample is used to compare the effect of the difference in the detector core circuit, and the individual detectors are considered under the optimal detection performance conditions [23]. Figure 13b shows the image obtained using the model capacitor, which is supported by the process design kit (PDK). Figure 13c shows an image obtained from a detector designed to achieve optimal detection performance using a customized capacitor in the same core circuit structure. The proposed detector, as shown in Figure 14, exhibited a 59.37% match with the normalized image, whereas previous studies demonstrated correlations of 41.4% and 53.7%. The concurrent-mode detector containing the cross-coupled capacitors better resolves the inner plus-shaped copper foil, which was impossible to identify in previous studies. The image results show that the image SNR of a single frame is 49 dB, which is 9 dB higher than that of the image obtained using the previous detector in one frame.

## 4. Conclusions

A CMOS detector with concurrent in-phase coupling was proposed to achieve high-quality images using THz imaging systems. The cross-capacitor structure possessed two detecting operations in a common source and a drain input structure while considering general amplifier analysis. The simulation results demonstrated that the proposed detector exhibits higher detection performance than the previously studied detector topology using gate-drain capacitors. At the output stage, the detector performance with regard to phase coupling was improved by 1.5–3 times higher than that of the previous detector core based on the input power. The detector, manufactured by a TSMC 0.25 μm CMOS process, comprised a differential folded dipole antenna, as the proposed core was connected by cross-coupled capacitors, a pre-amplifier, and a low-gain voltage buffer amplifier. The values of *R_V_* and *NEP* at 200 GHz were 14.13 MV/W and 34.42 pW/√Hz, respectively, at a gate bias of 150 mV. In contrast to the previous detector studies, the proposed detector structure has a smaller detector IC area with higher detection performance. At 200 GHz, the measurements of a THz imaging system using samples of copper foil tape attached to Styrofoam substrates demonstrated that the proposed detector can resolve wavelengths (approximately 200 GHz) of 2-mm thickness with a high correlation coefficient. The proposed detector demonstrated an improved correlation of 59.37% with the actual sample, 1.4 times higher than the previous detector under identical conditions, except for the circuit structure. The image SNR, which indicates the image quality, was 49 dB, which was 9 dB higher than that obtained using the model capacitor of the process. The THz image quality was improved using the proposed concurrent-mode CMOS detector without the need for an additional circuit.

## Figures and Tables

**Table 1:** Comparisons of CMOS detector performance.

Ref.	Process (nm)	Freq. (GHz)	Detector Core Configuration	*R_V_* (kV/W)	*NEP* (pW/√Hz)
[13]	250	200	Gate-drain cap.	5696	62.4
[16]	65	310	Drain input	2	3.5 ^1^
[19]	250	200	Gate-drain cap.	357.1	57.3
[22]	250	200	Gate-drain cap.	2020	76
[23]	90	365	Gate source input	1200	200
[24]	250	200	Gate-drain cap.	2990	46.3
This works	250	200	Concurrent-mode	14,130	34.42

^1^ Measured in a Faraday cage.

## Data Availability

The data presented in this study are available on request from the corresponding author. The data are not publicly available due to the regulation of the project.

## References

[1] Hu B.B., Nuss M.C. (1995). Imaging with terahertz waves. Opt. Lett..

[2] Hillger P., Grzyb J., Jain R., Pfeiffer U.R. (2019). Terahertz imaging and sensing applications with silicon-based technologies. IEEE Trans. Terahertz Sci. Technol..

[3] Kim J., Yoon D., Yun J., Song K., Kaynak M., Tillack B., Rieh J.-S. (2018). Three-Dimensional Terahertz Tomography with Transistor-Based Signal Source and Detector Circuits Operating Near 300 GHz. IEEE Trans. Terahertz Sci. Technol..

[4] Yun J., Oh S.J., Song K., Yoon D., Son H.Y., Choi Y., Huh Y.-M., Rieh J.-S. (2017). Terahertz reflection-mode biological imaging based on InP HBT source and detector. IEEE Trans. Terahertz Sci. Technol..

[5] Kawase K., Ogawa Y., Watanabe Y., Inoue H. (2003). Non-destructive terahertz imaging of illicit drugs using spectral fingerprints. Opt. Express.

[6] Liu H.-B., Zhong H., Karpowicz N., Chen Y., Zhang X.-C. (2007). Terahertz spectroscopy and imaging for defense and security applications. Proc. IEEE.

[7] Mansourzadeh S., Damyanov D., Vogel T., Wulf F., Kohlhass R.B., Globisch B., Hoffmann M., Balzer J.C., Saraceno C.J. (2021). High-power lensless THz imaging of hidden objects. IEEE Access.

[8] Rieh J.-S. (2021). Introduction to Terahertz Electronics.

[9] Ali M., Perenzoni M., Stoppa D. (2016). A methodology to measure input power and effective area for characterization of direct THz detectors. IEEE Trans. Instrum. Meas..

[10] Dyakonov M., Shur M.S. (1993). Shallow water analogy for a ballistic field effect transistor: New mechanism of plasma wave generation by DC current. Phys. Rev. Lett..

[11] Knap W., Teppe F., Meziani Y., Dyakonova N., Lusakowski J., Boeuf F., Skotnicki T., Maude D., Rumyantsev S., Shur M.S. (2004). Plasma wave detection of sub-terahertz and terahertz radiation by silicon field-effect transistors. Appl. Phys. Lett..

[12] Kojima H., Asano T. (2019). Impact of subthreshold slope on sensitivity of square law detector for high frequency radio wave detection. Jpn. J. Appl. Phys..

[13] Lee H.-J., Han S.-T., Yang J.-R. (2020). CMOS plasmon detector with three different body-biasing MOSFETs. IEEE Access.

[14] Kojima H., Kido D., Kanaya H., Ishii H., Maeda T., Ogura M., Asano T. (2019). Analysis of square-law detector for high-sensitive detection of terahertz waves. J. Appl. Phys..

[15] Sengupta K., Seo D., Yang L., Hajimiri A. (2015). Silicon integrated 280 GHz imaging chipset with 4 × 4 SiGe receiver array and CMOS source. IEEE Trans. Terahertz Sci. Technol..

[16] Shaulov E., Jameson S., Socher E. (2021). A zero bias J-band antenna-coupled detector in 65-nm CMOS. IEEE Trans. Terahertz Sci. Technol..

[17] Chai S., Lim S., Hong S. (2014). THz detector with an antenna coupled stacked CMOS plasma-wave FET. IEEE Microw. Wirel. Compon. Lett..

[18] Khan M.I.W., Kim S., Park D.-W., Kim H.-J., Han S.-K., Lee S.-G. (2018). Nonlinear analysis of nonresonant THz response of MOSFET and implementation of a high-responsivity cross-coupled THz detector. IEEE Trans. Terahertz Sci. Technol..

[19] Lee H.-N., Lee H.-J., Han S.-T., Yang J.-R. (2021). Highly sensitive CMOS plasmon detector with a low-gain buffer amplifier for terahertz imaging system. Microw. Opt. Technol. Lett..

[20] Han S.-T. (2020). Application of a compact sub-terahertz gyrotron for non-destructive inspections. IEEE Trans. Plasma Sci..

[21] Lee M.-J., Lee H.-N., Lee H.-J., Lee G.-E., Son J.-H., Yang J.-R. (2020). Analysis of the effects of differential integrated antennas on voltage responsivity of sub-terahertz CMOS detectors. IDEC J. Integr. Circuits Syst..

[22] Yang J.-R., Han S.-T., Baek D. (2017). Differential CMOS sub-terahertz detector with subthreshold amplifier. Sensors.

[23] Karolyi G., Gergelzi D., Foldesy P. (2014). Sub-THz sensor array with embedded signal processing in 90 nm CMOS technology. IEEE Sens. J..

[24] Lee G.-E., Lee H.-J., Han S.-T., Yang J.-R. (2021). CMOS detector using customized bolt-wrench capacitor on backend oxide layer. IEEE Microw. Wirel. Compon. Lett..

